# Joubert Syndrome: A Molar Tooth Sign in Disguise

**DOI:** 10.7759/cureus.9718

**Published:** 2020-08-13

**Authors:** Likhita Shaik, Abhimanyu Ravalani, Shruti Nelekar, Vamsi Krishna Gorijala, Kaushal Shah

**Affiliations:** 1 Internal Medicine, Ashwini Rural Medical College Hospital and Research Centre, Solapur, IND; 2 Medical Oncology, Mayo Clinic and Foundation, Rochester, USA; 3 Internal Medicine, Baroda Medical College, Vadodara, IND; 4 Pediatrics, Grant Medical College, Mumbai, IND; 5 Neurology, Guntur Medical College, Guntur, IND; 6 Psychiatry, Griffin Memorial Hospital, Norman, USA

**Keywords:** ciliopathy, joubert syndrome, molar tooth sign

## Abstract

Joubert syndrome (JS) is a rare genetic ciliopathy characterized by the aplasia or malformation of the midbrain and or hindbrain structures. It usually manifests during the early stages with nonspecific neurological symptoms that progress to involve multiple systems. Its presentation similarity to other neurological disorders makes the diagnosis difficult, hence causing a delay in treatment and worse prognosis due to complications. If undiagnosed during childhood, it often presents during adolescence with the most common complication of acute kidney injury due to nephronophthisis. Here, we present a case of JS in late adolescence with renal complications and other neurological abnormalities. We aim to emphasize the importance of its early diagnosis by physicians in childhood to prevent further complications. It also highlights the possible diagnostic value and significance of brain imaging in the early stages when only mild mental retardation signs may be the only clues.

## Introduction

Joubert syndrome (JS) is a rare neurodevelopmental disorder that was first described by Dr. Marie Joubert and her colleagues in 1969 [1]. It can be either an autosomal recessive or X-linked inherited ciliopathy. It is found in patients with one or more of 34 known pathological JS gene variants [2]. The products of these genes are localized in and around the primary cilium, which is associated with producing several molecules that play an essential role in the signaling pathways for the normal healthy development of the body organs, such as the brain, kidneys, liver, skeleton, and retina [3]. The primary cilia dysfunction, also known as ciliopathy, is caused due to abnormal genes. Malformation in the cerebellum and brainstem axis leads to defects in cognition, gait, speech, and ocular movements [3,4]. These signs and symptoms usually appear early in life and considered the prime clues for JS [5]. Other manifestations include congenital hepatic fibrosis and abnormal respiratory pattern, independent of age at presentation [5,6]. Renal dystrophy, cystic renal disease, skeletal abnormalities, and endocrine abnormalities usually present during the later stages of life [2,7,8]. We present a case of JS in late adolescence with deficiencies in renal and neurological functions.

## Case presentation

**Figure 1:**
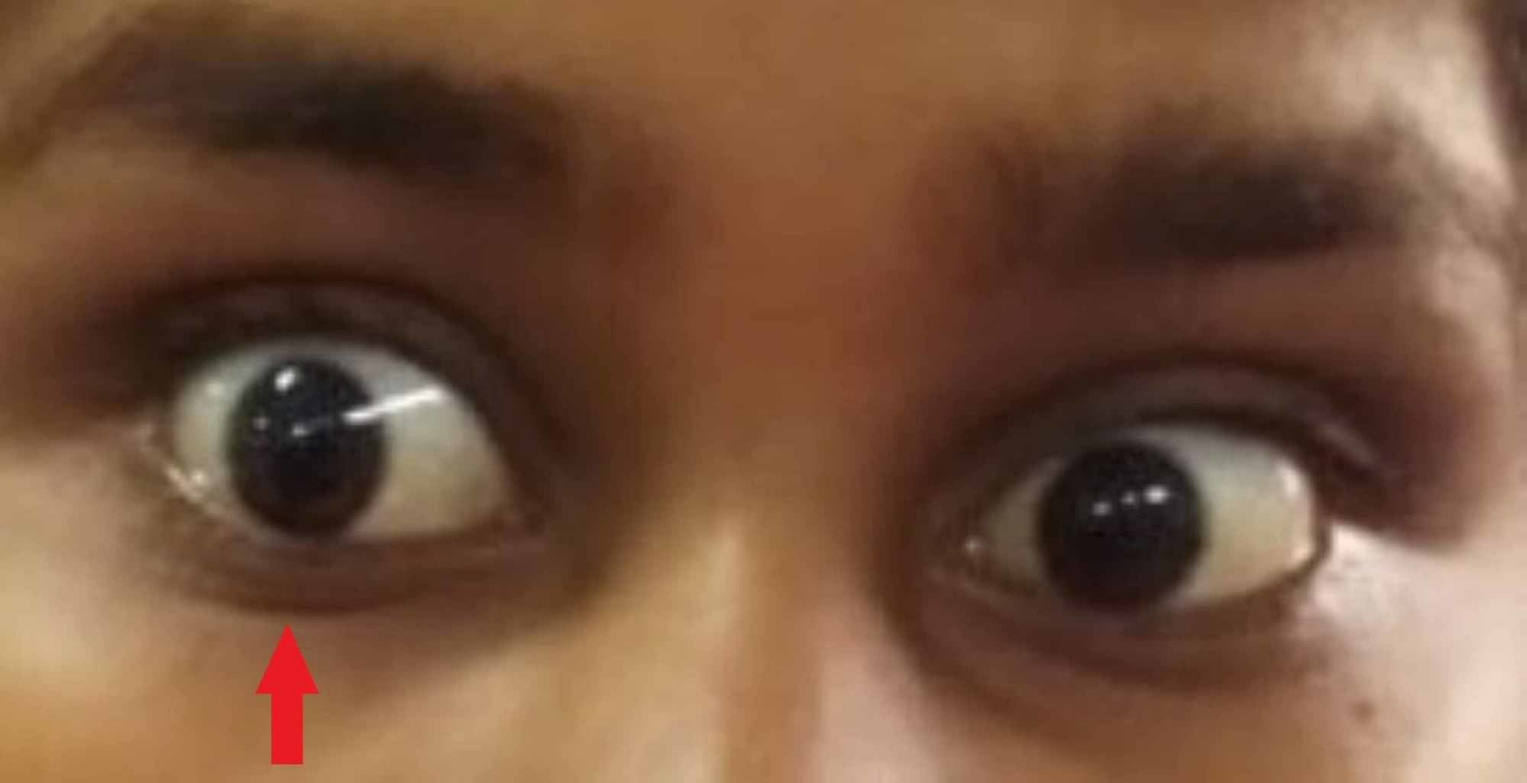
Right eye exotropia

A 16-year-old, young female patient presented to the pediatric emergency department with breathlessness, vomiting, decreased urinary output, and intermittent fever for the past seven days. On examination, the patient was alert, oriented to time place and person, and in mild distress due to dyspnea. Vital signs showed a heart rate of 100 beats per minute, blood pressure reading of 140/90 mm Hg, respiratory rate of 21, and oxygen saturation of 97%. Motor strength was 5/5 in upper extremities and 4/5 in lower extremities. Further examination showed no abnormalities, except for truncal ataxia. Eye examination found lid lag and nonparalytic exotropia of the right eye (Figure 1). 

The patient was born after an uneventful pregnancy at term via normal vaginal delivery. Mental retardation diagnosed since childhood, and all her developmental milestones were delayed by a year. The patient had also developed unsteadiness of gait about two months before presenting to the hospital. There was no history of any disease that could account for her current symptoms.

Investigations at admission revealed normocytic and normochromic anemia with hemoglobin of 7.3 g/dL (normal range: 12-15 g/dL). Her serum creatinine was 10.4 mg/dL (normal range: 0.5-1.1 mg/dL), and blood urea nitrogen level was 110 mg/dL (normal range: 8-21 mg/dL). Hyponatremia and hypokalemia were present with serum sodium and potassium values of 133 mEq/L (normal range: 135-145 mEq/L) and 3.3 mEq/L (normal range: 3.5-5 mEq/L), respectively. The urine analysis showed a 24-hour urinary protein excretion of 1,200 mg (normal: 150/24 hours). Her arterial blood gas analysis revealed an anion gap of 18 mEq/L (normal range: 8-16 mEq/L) with metabolic acidosis at the time of admission, with a pH of 7.31 (normal range: 7.35-7.45), serum bicarbonate of 4.9 mEq/L (normal range: 8-22 mEq/L), and pCO_2_ level at 9.9 mm Hg (normal range: 35-45 mm Hg). Urine and blood samples for culture and sensitivity testing were negative for infection.

**Figure 2:**
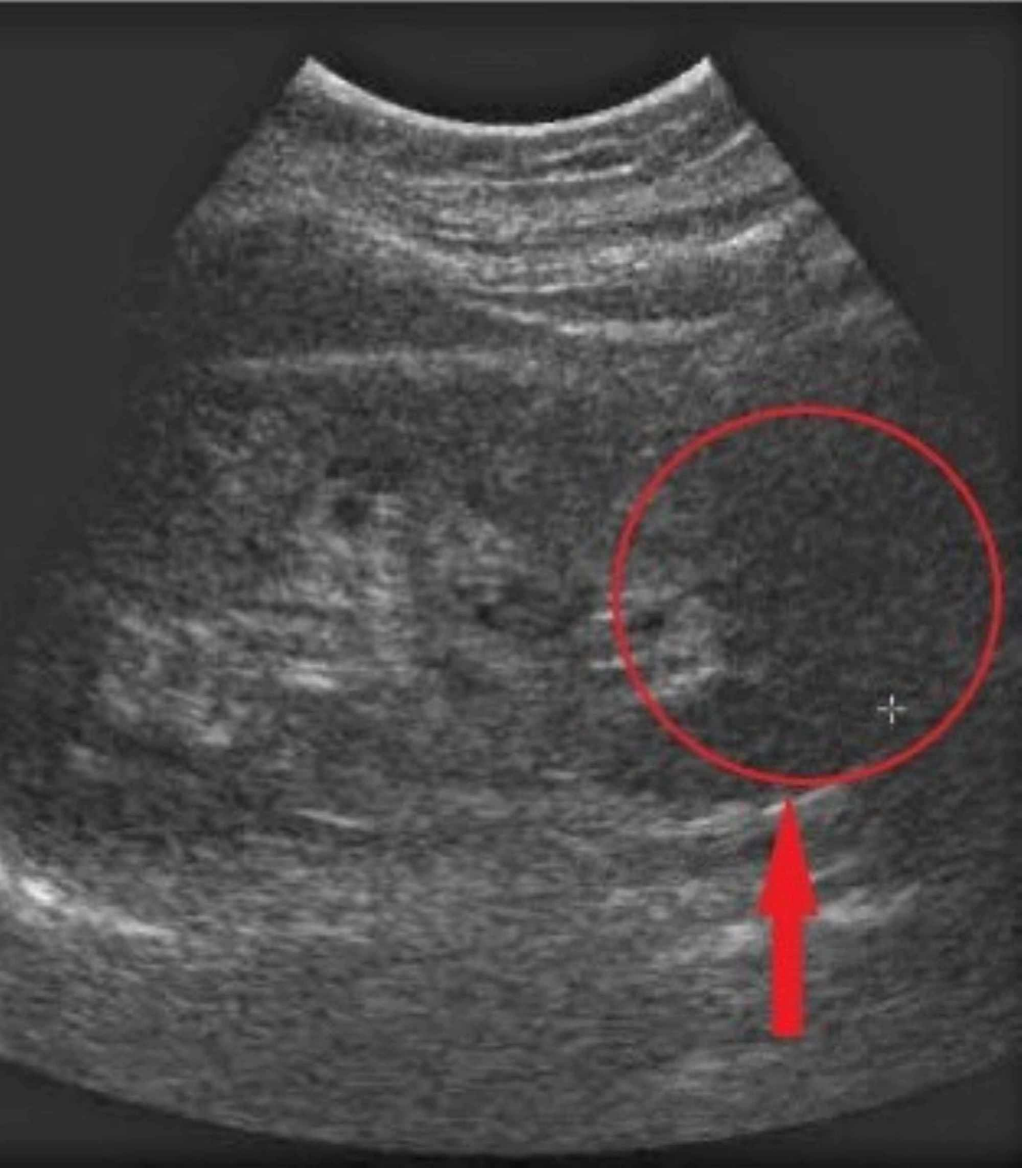
Cortical echogenicity of the renal parenchyma

The patient was hospitalized immediately, and intravenous (IV) fluids were initiated to correct the electrolyte imbalance. Abdominal ultrasound showed bilaterally contracted kidneys with raised cortical echogenicity of the renal parenchyma (Figure 2). 

**Figure 3:**
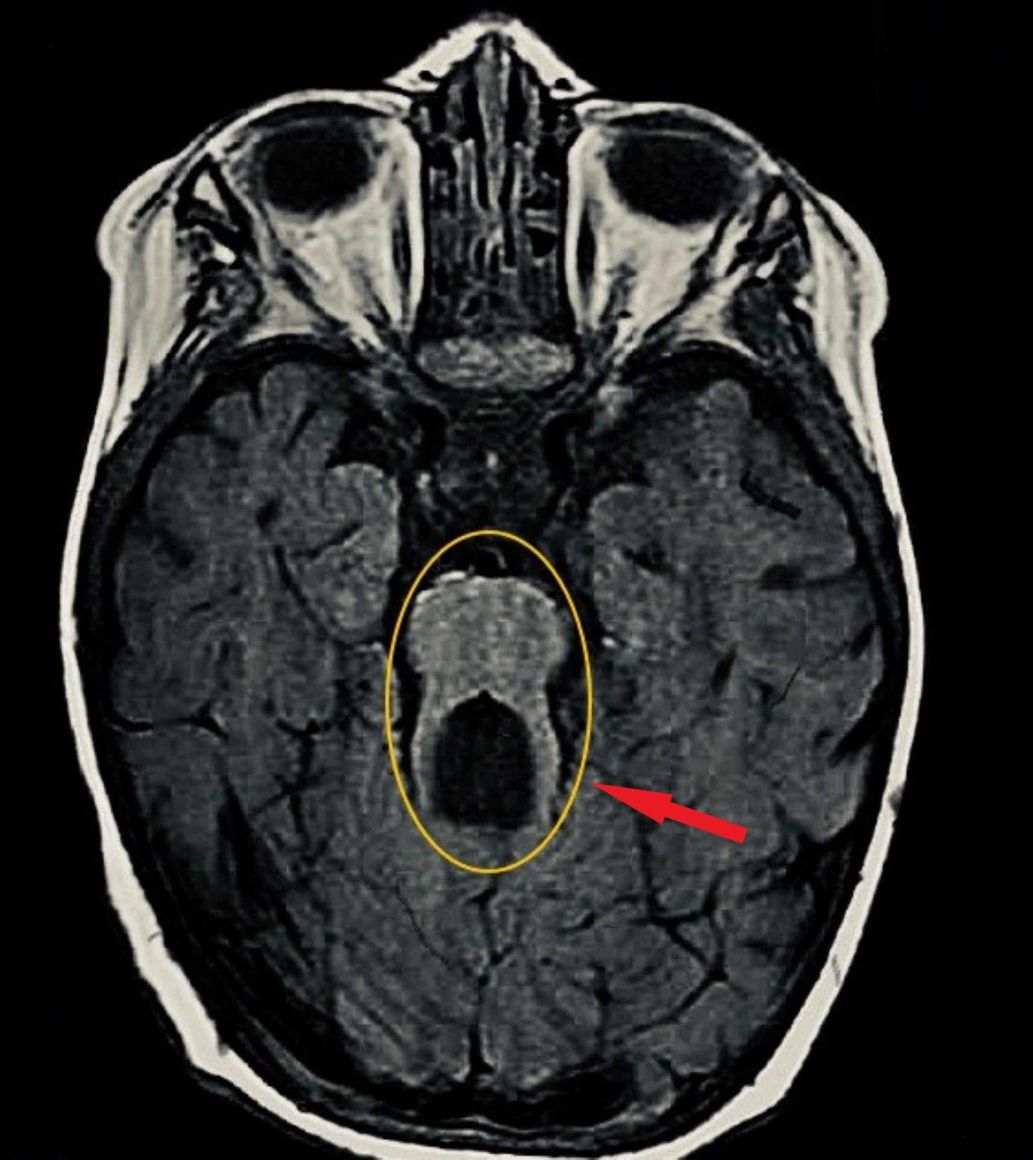
Classic sign of molar tooth seen in MRI of the brain

MRI of the brain was highly suggestive of JS, as it demonstrated a deepened interpeduncular cistern, thickened and elongated superior cerebellar peduncles bilaterally, with non-visualization of the cerebellar vermis, together creating a classic "molar tooth" appearance (Figure 3).

Clinical presentation and imaging and laboratory are suggestive of JS with chronic kidney disease. A central venous catheter through a subclavian vein and hemodialysis was initiated along with medications. The patient was started on antibiotics via oral norfloxacin 800 mg/day and IV meropenem 1,500 mg/day and metronidazole 1,500 mg/day. An IV proton pump inhibitor of pantoprazole 40 mg/day was initiated to prevent gastrointestinal side effects from antibiotics. Antipyretic acetaminophen two cubic centimeters (cc) and an antiemetic ondansetron 4 mg were injected as needed. The patient was also started on stool softener lactulose 30 g at bedtime and oral vitamin supplements of folic acid 10 mg/day and calcium lactate 600 mg/day. 

After nine days of admission and seven hemodialysis sessions, the patient showed clinical improvement, along with a decrease in serum creatinine value to 4.5 mg/dL and an increase in hemoglobin level to 10.6 g/dL. Her repeat serum electrolyte levels and blood gas analysis showed a return to values within the normal range. The patient's parents were counseled on the daily nutritional intake to improve muscle mass and to follow up with neurology and nephrology to evaluate her condition further. Lastly, she was discharged from the hospital with oral cefixime 400 mg/day, norfloxacin 800 mg/day, furosemide 40 mg/day, spironolactone 100 mg/day, lactulose 30 g/day, oral folic acid 10 mg/day, and calcium lactate 600 mg/day. 

## Discussion

JS is a rare genetic disorder with a prevalence of one in 80,000 to 100,000 live births [9]. Pathology pertaining to it rests in the midbrain and hindbrain malformation, which is represented on CT or MRI as the "molar tooth sign" due to malformation of cerebellar vermis and brainstem. It usually presents nonspecific symptoms such as hypotonia, atonia, and weakness, which evolve during the course into ataxia, apnea-hyperpnea, gait abnormalities, delayed milestones, and pathological ocular movements. Coarse facial features like broad forehead, arched eyebrows, prognathia, ptosis, hypertelorism, low set ears, and a trapezoid-shaped mouth are typical [5,10]. 

Genes such as NPHP1, AHI1, CEP290, RPGRIP1L, TMEM67, MKS3, ARL13B, and CC2D2A have been known to play a causal role in its pathogenesis. They play a vital role in the functioning of the cilium and basal organelle; hence it is termed "ciliopathies" [11]. Since cilia is an essential component in the functioning of the brain, kidneys, retina, and other organs, its presentation during later ages as multiorgan failure is not uncommon [12]. 

Various conditions such as COACH (Cerebellar vermis hypo/aplasia, Oligophrenia, congenital Ataxia, Coloboma and Hepatic fibrosis) syndrome, Senior-Loken syndrome, Varadi-Papp syndrome, and nephronophthisis also present signs and symptoms similar to JS, which often mislead the diagnosis. It is often unclear whether the conditions are variants of the same or completely different disorders [9]. Even classical presentations seem to be nonspecific enough to confuse the clinicians with other likely disorders. The diagnostic approach after clinical suspicion should be to rule out reversible causes and proceed towards imaging. Demonstration of mesial temporal sclerosis on MRI is usually sufficient to diagnose a JS case, but in cases with unclear MRI, diffusion tensor imaging (DTI) can assist the diagnosis by the clear demarcation of absent crossing fibers at the midbrain level [13]. The treatment of the JS includes the management of its affected areas. A diagnostic workflow and regular physical examinations need to be performed once the diagnosis is established to prevent complications and deterioration of symptoms. Early respiratory symptoms should be addressed by assisted ventilation, though a majority subside with age. Efficient rehabilitation, especially neuro-ophthalmological techniques, helps overcome the burden of neurological and psychiatric complications like ocular apraxia, mental retardation, delayed milestones, and abnormal cognition [14]. Nephrology studies and renal replacement measures carry the utmost value in early detection and treatment of complications, especially in adolescents where cystic malformation in kidneys in the form of juvenile nephronophthisis is a common presentation. Other signs and symptoms may mask renal issues, thus presenting as acute or chronic renal insufficiency in later stages of JS [2]. Congenital liver fibrosis could be another presenting feature during later adulthood. In a nutshell, the management relies on the early diagnosis of the condition and treatment of its complications. The initial evidence on ultrasonography during the second or third trimester of pregnancy may provide a lead in time and prolong the life expectancy. Hence, high-frequency carrier populations such as the Mediterranean need to be screened for better outcomes [15].

## Conclusions

JS remains one of the rarest conditions found clinically and not often discussed in the literature. Diagnostic imaging such as MRI and DTI are keys to differentiating JS from other related disorders during early stages. The timing of diagnosis plays a crucial role in preventing complications and reducing morbidity. Appropriate supportive and symptomatic measures help to improve the condition temporarily and quality of life. However, routine follow-ups with relevant specialties, such as neurology, nephrology, and gastroenterology, help maintain the equilibrium of the condition and reduce mortality rates through acute presentations that are not always treatable. A definite need for more definitive treatment necessitates further research on this disease.
